# DNA Metabarcoding and Isolation by Baiting Complement Each Other in Revealing *Phytophthora* Diversity in Anthropized and Natural Ecosystems

**DOI:** 10.3390/jof8040330

**Published:** 2022-03-22

**Authors:** Federico La Spada, Peter J. A. Cock, Eva Randall, Antonella Pane, David E. L. Cooke, Santa Olga Cacciola

**Affiliations:** 1 Department of Agriculture, Food and Environment, University of Catania, 95123 Catania, Italy; federico.laspada@unict.it (F.L.S.); apane@unict.it (A.P.); 2 The James Hutton Institute, Invergowrie, Dundee DD2 5DA, UK; peter.cock@hutton.ac.uk (P.J.A.C.); eva.randall@hutton.ac.uk (E.R.)

**Keywords:** leaf baiting, DNA sequencing, eDNA, metabarcoding, Illumina, ASVs, rhizosphere soil, new species, detection, nature reserve, botanical garden, citrus orchards

## Abstract

Isolation techniques supplemented by sequencing of DNA from axenic cultures have provided a robust methodology for the study of *Phytophthora* communities in agricultural and natural ecosystems. Recently, metabarcoding approaches have emerged as new paradigms for the detection of *Phytophthora* species in environmental samples. In this study, Illumina DNA metabarcoding and a conventional leaf baiting isolation technique were compared to unravel the variability of *Phytophthora* communities in different environments. Overall, 39 rhizosphere soil samples from a natural, a semi-natural and a horticultural small-scale ecosystem, respectively, were processed by both baiting and metabarcoding. Using both detection techniques, 28 out of 39 samples tested positive for *Phytophthora*. Overall, 1,406,613 *Phytophthora* internal transcribed spacer 1 (ITS1) sequences and 155 *Phytophthora* isolates were obtained, which grouped into 21 taxa, five retrieved exclusively by baiting (*P. bilorbang; P. cryptogea; P. gonapodyides; P. parvispora* and *P. pseudocryptogea*), 12 exclusively by metabarcoding (*P. asparagi; P. occultans; P. psycrophila; P. syringae; P. aleatoria*/*P. cactorum; P. castanetorum*/*P. quercina; P. iranica*-like; *P.* unknown sp. 1; *P.* unknown sp. 2; *P.* unknown sp. 3; *P.* unknown sp. 4; *P.* unknown sp. 5) and four with both techniques (*P. citrophthora*, *P. multivora*, *P. nicotianae* and *P. plurivora*). Both techniques complemented each other in describing the variability of *Phytophthora* communities from natural and managed ecosystems and revealing the presence of rare or undescribed *Phytophthora* taxa.

## 1. Introduction

Among plant pathogens threatening crops and natural ecosystems, oomycetes of the *Phytophthora* genus stand out with around 200 recognized species [1,2]. This genus includes species well-known for their ability to impact plant ecosystem stability and reduce the productivity of crops on a global scale [3,4,5,6,7,8,9,10]. Most *Phytophthora* species have a very broad host range, including *P. cinnamomi* [11,12,13,14]; *P. multivora* [15,16]; *P. nicotianae* [17,18,19,20,21]; *P. niederhauserii* [22] and *P. ramorum* [23,24,25,26], whereas others are only isolated from a restricted host-plant range, such as *P. ilicis* [27], the recently described *P. oleae* [28] and the well-known *P. infestans* [29], respectively. Conversely, a single host plant species may be infected by many *Phytophthora* species, as exemplified by the cases of *Citrus* species and olive (*Olea europaea*) [2,28,30,31,32,33].

For the past two decades, molecular diagnostic tools for the accurate identification of cultured *Phytophthora* species together with established isolation techniques have provided a robust step-by-step methodology for the surveillance of *Phytophthora* communities from natural, semi-natural and horticultural ecosystems [34,35,36,37]. Among isolation techniques, baiting is a widely used method for the recovery of *Phytophthora* from rhizosphere soil samples of managed and natural ecosystems. In particular, leaf baiting has been used successfully to assess *Phytophthora* diversity in natural ecosystems [6,8,37,38,39,40], nurseries [41], public and botanical gardens [42] as well as horticultural crops [43]. However, despite its theoretical simplicity, several crucial aspects can affect the outcome of the soil baiting process. The behavioral traits and biological features of the infective propagules (zoospores), together with the differences in physiology and ecology between species of *Phytophthora,* are potential challenges. In particular, careful use of appropriate baits and the compliance with strict technical times and thermo-hygrometric conditions are required [44]. Consistent isolation of only *Phytophthora* from soil samples that contain thousands of other organisms, including other oomycetes, involves significant technical skill and experience. The success of baiting is affected by several factors: the seasonality and type of propagules in the sample [45]; the variability of climatic conditions; the inability to culture obligate and biotrophic species [46,47] and the presence of dead or resting propagules which go undetected with this type of assay.

In general, sequencing of DNA from axenic cultures obtained by baiting or direct isolation from infected tissues or soil has provided clearer insights into the knowledge of the diversity of fungal and oomycete pathogens and several distinct species of the same genus were shown to be associated with plant diseases that had initially been considered to be caused by a single pathogen. Examples include olive anthracnose, pomegranate heart rot, sweet basil black spot and the well-known sudden oak death [48,49,50,51,52,53,54,55]. However, not all the identified species were equally aggressive and, in most cases, a few prevailed over the others.

Metabarcoding approaches are emerging as new paradigms for the detection of all the species of a target genus present within an environmental sample [56,57,58,59]. The *Phytophthora* diversity from environmental samples has been studied to date using three different sequencing technologies: (i) a conventional cloning Sanger/sequencing (CSS) method applied to environmental DNA (eDNA) from rhizosphere soil and roots from ornamental and fruit nursery plants [60]; (ii) 454 Pyrosequencing, to assess the diversity from forest ecosystems [45,46,61,62,63,64] and nurseries [58]; and (iii) Illumina technology for the characterization of *Phytophthora* diversity from plants in public gardens/amenity woodlands [1], plant nurseries [59] and declining holm oak forests [65]. Metabarcoding studies are currently considered a useful tool for routine surveillance aimed at early detection of invasive pathogens [1]. Furthermore, studies which compared outcomes of metabarcoding with those of baiting indicated a greater resolution from metabarcoding over the conventional baiting methods in environmental analyses of *Phytophthora* species diversity [1,45,46,64]. However, as for baiting, metabarcoding techniques are also affected by various limitations, such as the inability to generate pure cultures that are crucial for taxonomic and genomics studies as well as for quarantine purposes, the inability to discriminate among some closely related species [46] and false positives due to the presence of dead propagules. The latter is an inherent limit of culture-independent detection methods [66]. Another important issue seems to influence the metabarcoding results, namely the nature of the processed samples. Discrepancies were observed in the qualitative outcomes from different matrices (roots, soil and mixtures of soil and roots) in the same environmental sample [60,61,62,63,64], suggesting an uneven distribution of propagules of the target organism in the original sample. In order to test the above hypotheses and to compare the efficacy and limits of traditional soil baiting and Illumina metabarcoding to understand the diversity of soil-borne *Phytophthora* communities in different ecosystems, both methods were used to analyze rhizosphere samples, including both soil and fine roots, collected from different ecosystems. In particular, the aims of this study were: i. to test whether Illumina metabarcoding of eDNA can reveal differences in the species diversity of *Phytophthora* in different environmental matrices (roots, soil and mixtures of soil and roots) of the same sample; ii. to compare the efficacy and limits of traditional soil baiting and Illumina metabarcoding to understand the variability of soil-borne *Phytophthora* communities in different types of small-scale ecosystem characterized by increasing levels of anthropization: (i) a nature reserve; (ii) a botanical garden; and (iii) a managed commercial citrus orchard.

## 2. Materials and Methods

### 2.1. Sampling Areas and Collection of Rhizosphere Soil Samples

**Figure 1:**
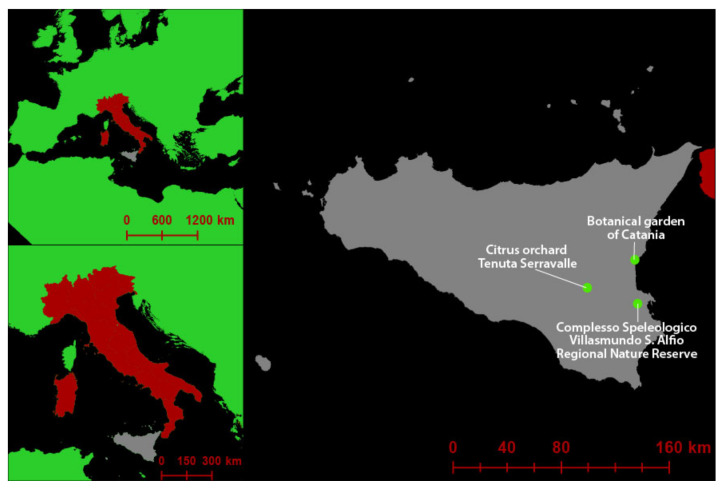
Geographical location of the three surveyed areas included in this study.

Three sampling sites in the eastern region of the Mediterranean island of Sicily (Italy) were selected for this study: (i) a nature reserve (Complesso Speleologico Villasmundo S. Alfio Regional Natural Reserve—Melilli, Siracusa, Italy); (ii) the botanical garden of the University of Catania, (Catania province); and (iii) a 40-year old commercial citrus orchard of sweet orange (*Citrus* × *sinensis* ‘Tarocco’) on sour orange (*C.* × *aurantium*) as rootstock (Tenuta Serravalle, Mineo, Catania province). Sampling activities were carried out during the spring (March–May) of 2019 (Figure 1).

In total, 39 rhizosphere soil samples were collected from the selected sampling areas (Table 1): (i) 17 samples from both tree and herbaceous plants of the main vegetation types of the reserve, which extended to ca. 71.7 ha; (ii) 12 samples from ornamental trees in the botanical garden; and (iii) ten samples from citrus trees in a plot of ca. 2.5 ha inside the orchard which extended overall ca. 65 ha.

Soil sampling was performed in accordance with Jung et al. [8] with the following modifications: at each tree, the organic litter layer was removed; then, three rhizosphere soil monoliths (ca. 350 g each), containing fine roots, were taken by means of a spade at a depth of about 20–40 cm from the base of the stem; the three monoliths were then thoroughly mixed and bulked to a final sample of ca. 1 kg.

**Figure 2:**
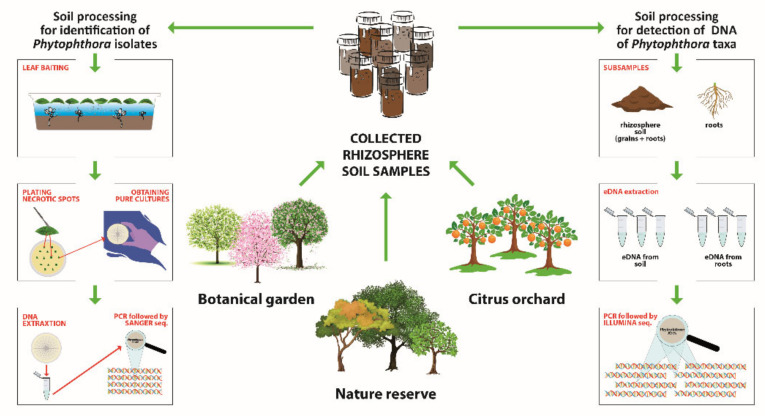
Experimental design of the study.

For each final sample, an aliquot of ca. 200–250 g containing fine roots and soil was separated, immediately frozen in liquid nitrogen and stored at −80 °C until use for metabarcoding analyses. The remaining rhizosphere soil (soil associated with fine roots) was used for the traditional isolation of *Phytophthora* spp. by baiting. The complete experimental design of the study is illustrated in Figure 2.

### 2.2. DNA Extraction from Rhizosphere Soil Samples for Metabarcoding Analyses

For each rhizosphere soil sample, eDNA extractions were carried out in triplicate using a published CTAB method [67] with slight modifications. Briefly, triplicate soil subsamples (40 g each) were suspended in 80 mL of CTAB-PO_4_ Buffer (21.4 g/L Na_2_HPO_4_, 20.0 g/L CTAB (Hexadecyltrimethylammonium Bromide), 87.7 g/L NaCl; pH 8.0) and milled for 5 min at 300 rpm in a Planetary Ball Mill (Retsch, PM 400; Retsch GmbH, Haan, Germany) in the presence of 12 stainless steel ball bearings (2 cm diameter).

For each subsample, a 1.5 mL aliquot (equivalent to 0.50 g soil) from the obtained soil suspension was transferred to a 1.5 mL Eppendorf tube. Samples were then centrifuged at 6000 rpm and the supernatant was thoroughly mixed with an equivalent volume of cold chloroform, twice briefly vortexed and re-centrifuged (13,000 rpm × 4 min). The eDNA in the aqueous phase was then precipitated with 90 μL of a 3 M solution of sodium acetate and an equal volume of isopropanol for 1 h at room temperature. The eDNA was pelleted by centrifugation (13,000 rpm for 4 min), washed with 150 μL of 70% ethanol, re-pelleted and then re-suspended in 100 μL of 1X TE buffer (10 mM Tris–HCl and 1 mM EDTA; pH 8.0) and stored at −20 °C until required. Soil eDNA extract obtained from each sample was then purified by centrifugation (12,300 rpm × 1 min) in a Micro Bio-Spin column (Bio-Rad Laboratories, Hercules, CA, USA) filled with water-insoluble PVPP (polyvinylpolypyrrolidone).

In addition, eDNA extractions from each collected rhizosphere soil sample were also performed in three further subsamples comprising mainly fine roots hand-picked from the main samples. For this extraction, samples were crushed to a fine powder with liquid nitrogen and processed with the PowerSoil^®^ DNA Isolation Kit (MoBio Laboratories Inc., Carlsbad, CA, USA) or the FastDNA^®^ SPIN Kit for Soil (MP Biomedicals, Santa Ana, CA, USA), in accordance with the respective manufacturer’s instructions.

Overall, following both extraction methods 195 eDNA samples were obtained which were then processed further.

### 2.3. PCR Reactions

To provide the required sensitivity, a nested PCR to amplify a ~250 base pair fragment of the internal transcribed spacer 1 (ITS1) region of the nuclear ribosomal DNA (rRNA gene) was performed in accordance with the protocol of Riddell et al. [1] by using primer pairs 18Ph2F (5′-GGATAGACTGTTGCAATTTTCAGT-3′) and 5.8S-1R (5′-GCARRGACTTTCGTCCCYRC-3′) [36] in the first round and ITS-6 (5′-GAAGGTGAAGTCGTAACAAGG-3′) [68] and 5.8S-1R in the second round. The second-round primers were amended with overhang adapters to ensure compatibility with the Illumina index and sequencing adapters. These were: forward overhang; 5′-TCGTCGGCAGCGTCAGATGTGTATAAGAGACAG-3′ (ITS-6) and reverse overhang; 5′-GTCTCGTGGGCTCGGAGATGTGTATAAGAGACAG-3′ (5.8S-1R) [69]. In each case hi-fidelity KAPA HiFi DNA Polymerase (F. Hoffmann–La Roche SA, Basel, Switzerland) was used. The amplicons obtained were then detected in 1.5% agarose gel and all *Phytophthora*-positive PCR products were pooled for downstream processing.

### 2.4. Illumina Sequencing Library Preparation and Sequencing

PCR products were prepared for the Illumina sequencing following the instructions reported in the protocol 16S Metagenomic Sequencing Library Preparation [69]. Briefly, obtained amplicons were subjected to a PCR clean-up by using the Agencourt^®^ Ampure^®^ XP beads kit (Agencourt Bioscience, Beverly, MA, USA) and then to Index PCR using the Nextera XT Index Kit (Epicentre, Madison, WI, USA) to attach dual indices and Illumina sequencing adapters to each amplicon. This step made the unique identification and distinction of each amplicon during the sequencing run possible. A second PCR clean-up was then run, as above, and the obtained final library from each sample was visualized in 1.5% agarose. All the libraries were then quantified by fluorimetry with the Quant-iT™ PicoGreen™ dsDNA Assay Kit (Invitrogen™, Waltham, MA, USA) and each was adjusted to 4 nM. Libraries were then sequenced at the James Hutton Institute (Dundee, United Kingdom) by using the MiSeq v. 2500 cycles reagent kit (MS-102-2003, Illumina, San Diego, CA, USA). The obtained FASTQ files containing reads for each sample were then exported for bioinformatic analysis.

### 2.5. Analyses of Illumina Data

The obtained reads from all the three sampling areas were grouped into unique Amplicon Sequence Variants (ASVs) using v0.6.1 of the sequence-based diagnostic/profiling Tree Health and Plant Biosecurity Initiative (THAPBI) *Phytophthora* ITS1 Classifier Tool (PICT) [70]. Based on unwanted *Phytophthora* ITS1 in the control samples, an unusually stringent minimum abundance threshold just over 2000 copies was used (meaning with a median of almost 60,000 reads per sample, accepted unique ITS1 sequences typically represent at least 3% of a sample). Species identification was based on the THAPBI PICT v0.6.1 *Phytophthora* ITS1 curated database and a local database containing sequences of ex-type or key isolates from published studies [8,30,38,68,71,72,73]. ASVs were assigned to a species when their sequences were at least 99% identical to a reference isolate. Any sequence not assigned to a species from the reference databases was compared to the GenBank nt database using BLASTN+ [74]; the best matches to *Phytophthora* were then subjected to phylogenetic analyses to show their position within the relevant ITS *Phytophthora* Clade (the software MEGAX [75] was used).

The *Phytophthora* diversity from Illumina-positive samples from the three sampling areas was calculated by using the Shannon Diversity index ((*H = −Σp_i_ln(p_i_)*), the Pielou’s evenness index (*J = H/lnS*) and the Simpson dominance index (*λ = 1/Σp_i_*^2^), where *p_i_* represents the frequency of each ASV and *S* the number of ASVs per sampling site. Since the assumption of normal distribution was violated (the Shapiro–Wilk test was applied), the statistical differences in the diversity among sampling areas were assessed by the Chi-square non-parametric test of Kruskal–Wallis followed by Dunn’s multiple comparison post-hoc test (the R software [76] was used).

### 2.6. Isolation by Baiting and Identification of Phytophthora Isolates

Isolations of *Phytophthora* specimens from rhizosphere soil samples were carried out in accordance with Jung et al. and Aloi et al. [8,77]. Within 24 h of the collection, an aliquot of ca. 250 g from each rhizosphere soil sample (soil associated with fine roots) was flooded with distilled water keeping the distance between the surface of the soil and the waterline at around 3–4 cm. Young and tender leaves from carob (*Ceratonia siliqua*) and oak (*Quercus ilex* and *Q. pubescens sensu latu*) were floated on the water surface of the flooded soil and incubated in the dark for 48 h at 25 °C. Any necrotic spots (2 × 2 mm) from the symptomatic leaves were then excised and plated on PARPNH V8-agar [7]. Petri dishes were incubated at 22 °C in the dark. Emerging *Phytophthora* hyphae were transferred onto V8-agar Petri plates under the stereomicroscope. All the single-hypha *Phytophthora* isolates were maintained on V8-agar in the dark at a temperature of 15 °C.

Seven-day-old cultures grown at 22 °C in the dark on V8-agar were used to group all isolates from each sampling site into morphotypes based on their growth pattern. Morphological features of sporangia; oogonia; antheridia; chlamydospores; hyphal swellings and aggregations were also checked (data not shown) [7] in relation to species already described in the literature [5,15,72,73,78,79]. Species’ identification was confirmed by the amplification and analysis of the Internal Transcribed Spacer (ITS) regions of the ribosomal DNA (rDNA). Total DNA was extracted from seven-day-old cultures grown on V8-agar at 20 °C using the DNeasy Plant Pro Kit (QIAGEN, Hilden, Germany) following the manufacturer’s instructions. PCR amplifications were performed using the *Taq* DNA polymerase recombinant (Invitrogen™, Waltham, MA, USA) with the universal primer pairs ITS-6 (5′-GAAGGTGAAGTCGTAACAAGG-3′) [68] and ITS-4 (5′-TCCTCCGCTTATTGATATGC-3′) [80]; each amplification was carried out in a 25 μL reaction mix containing PCR Buffer (1X), dNTP mix (0.2 mM), MgCl2 (1.5 mM), forward and reverse primers (0.5 μM each), *Taq* DNA Polymerase (1 U) and 100 ng of DNA. The thermocycler conditions were as follows: 94 °C for 3 min; followed by 35 cycles of 94 °C for 30 s, 55 °C for 30 s, and 72 °C for 30 s; and then 72 °C for 10 min.

The amplicons were detected in 1% agarose gel and sequenced in both directions by an external service (Macrogen, Seoul, South Korea). Sequences were analyzed using FinchTV v.1.4.0 [81] and MEGAX [75]. For species identification, consensus sequences were blasted against GenBank [82] and a local database containing sequences of ex-type or key isolates from published studies. Isolates were assigned to a species when their sequences were at least 99% identical to a reference isolate. ITS sequences from representative isolates of this study were deposited at GenBank 1 ([83]; accession numbers are reported in Table S1).

## 3. Results

### 3.1. Phytophthora Species Detected by Illumina Sequencing from Environmental DNA

A total of 40 replicate eDNA samples (13 from soil and 27 from roots) from 19 out of 39 collected rhizosphere soil samples (nine from the nature reserve, seven from the botanical garden and three from the citrus orchard) produced amplicons in the nested PCR. The Illumina sequencing run generated more than 2,000,000 high quality sequence reads taken forward for the bioinformatic analysis. Two CO samples failed Illumina sequencing, giving minimal yield. The data globally generated 1,503,919 accepted ITS1 sequences which could be grouped into 32 ASVs. Bioinformatic and phylogenetic analyses allowed the discrimination of 1,406,613 *Phytophthora*-reads which were organized in 24 *Phytophthora* ASVs that comprised (i) eight clearly distinguishable known species; (ii) two ASVs each of which was identical to a pair of closely related species that could not be discriminated using ITS1 barcodes; and (iii) six unknown *Phytophthora* taxa (Table S2). Among these, 12 were exclusively detected in eDNA from roots, one only in soil and three in both roots and soil (Table 1 and Table S2). Seven of the additional eight ASVs matched known or unknown species of downy mildew (*Plasmopora*, *Peronospora* and *Hyaloperonospora*) at 97 to 100% identities (Table S3). The last ASV was a single base pair variant of *P. psychrophila* found in a replicate of sample 8 from the nature reserve (Table S3). This replicate had an unusually high number of reads (over 138 K) with an exact match to *P. psychrophila* and it is probable that the variant, with around 2.5 K reads, reflects a PCR amplification or Illumina sequencing artefact (see Tables S2 and S3).

#### 3.1.1. Nature Reserve

Overall, ten *Phytophthora* taxa were detected in the reserve (Table 1). Among these, eight were detected exclusively in roots, one in both roots and soil (namely, *P. psychrophila*) and one exclusively in soil (namely, *P. castanetorum/P. quercina*) (Table 1).

**Figure 3:**
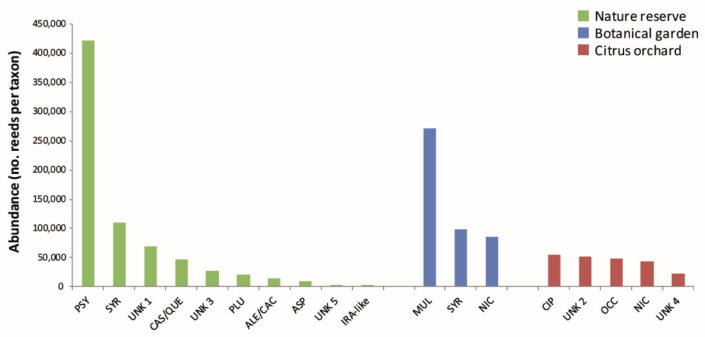
Read abundance of *Phytophthora* taxa identified in the three surveyed areas obtained by DNA metabarcoding. ALE/CAC = *P. aleatoria*/*P. cactorum*; ASP = *P. asparagi*; CAS/QUE = *P. castanetorum*/*P. quercina*; CIP = *P. citrophthora*; IRA = *P. iranica*-like; MUL = *P. multivora*; NIC = *P. nicotianae*; OCC = *P. occultans*; PLU = *P. plurivora*; PSY = *P. psychrophila*; SYR = *P. syringae*; UNK 1 = *Phytophthora unknown* sp. *1*; UNK 2 = *Phytophthora* unknown sp. 2; UNK 3 = *Phytophthora* unknown sp. 3; UNK 4 = *Phytophthora* unknown sp. 4; UNK 5 = *Phytophthora* unknown sp. 5.

With a total of 421,353 reads (Figure 3) occurring in six out of nine metabarcoding-*Phytophthora* positive soil samples (Table 1), *P. psycrophila* was the most abundant and widespread species within the reserve; its DNA barcode was recorded in samples from rhizosphere soil collected beneath trees (*Salix pedicellata*, *Platanus orientalis*, *Quercus ilex*, *Quercus pubescens sensu latu*) and annual herbaceous plants (*Cynaria cardunculus*) (Table 1).

Another species with a high detection frequency (records in four out of nine metabarcoding-*Phytophthora* positive soil samples, Table 1) was *P. syringae*, whose DNA was mainly reported from roots belonging to rhizosphere soils collected beneath trees of *Q. ilex*, *Q. pubescens s. l.* and *Pistacia lentiscus* (Table 1).

**Figure 4:**
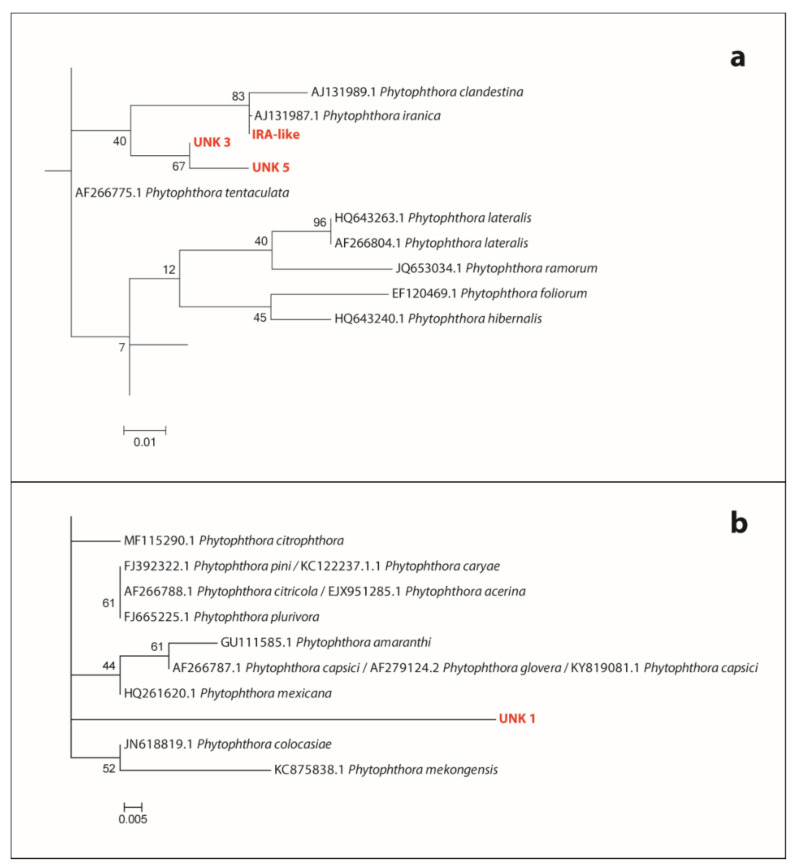
Sections from the phylogenetic tree of the *Phytophthora* ITS1 locus generated by the Maximum Likelihood method, based on the Tamura-Nei model. (**a**) Position of *Phytophthora iranica*-like (IRA-like) unknown sp.3 (UNK 3) and sp.5 (UNK 5) within *Phytophthora* clade 1a; (**b**) Position of *Phytophthora* unknown sp.1 within *Phytophthora* clade 2. The bootstrap consensus tree inferred from 1000 replicates is taken to represent the evolutionary history of the taxa analyzed. Branches corresponding to partitions reproduced in less than 50% bootstrap replicates are collapsed. The percentage of replicate trees in which the associated taxa clustered together in the bootstrap test (1000 replicates) is shown next to the branches.

Apart from the recovery of the DNA of well-known *Phytophthora* species in Mediterranean forests (viz. *P. asparagi* and *P. plurivora*), it is noteworthy that the detection from *Cynara cardunculus* roots of DNA barcodes shows 99.45% similarity to sequences of *P. iranica*; this ASV has been designated here as *P. iranica*-like. Barcode sequences of three unknown *Phytophthora* taxa, here designated as “*Phytophthora* unknown sp.” 1 (UNK 1), three (UNK 3) and five (UNK 5) were also detected in the reserve. Phylogenetic analyses placed *P. iranica*-like, UNK 3 and UNK 5 into the *Phytophthora* ITS clade 1a (Figure 4a) and UNK 1 in the *Phytophthora* ITS clade 2 (Figure 4b).

Finally, barcodes from the pairs of taxa that could not be discriminated, namely *P. aleatoria/P. cactorum* and *P. castanetorum/P. quercina*, were detected in the rhizosphere soil of *P. lentiscus* and *Q. ilex*, respectively (Table 1).

#### 3.1.2. Botanical Garden

*Phytophthora multivora*, *P. syringae* and *P. nicotianae* were detected in the samples from the botanical garden (Table 1). The DNA barcode of the most abundant and widespread species, *P. multivora* (records in five out of seven metabarcoding-*Phytophthora* positive rhizosphere soil samples, Table 1), was detected exclusively in the processed roots from rhizosphere soil samples collected beneath trees of *Grevillea robusta*, *Eucalyptus citridora*, *Olea europea*, *P. lentiscus* and in soil from *Pistacia atlantica*. *P. syringae* was detected in roots from *E. citridora* and *Q. suber* and *P. lentiscus* and in soil from *P. atlantica* (Table 1). Together with *P. multivora* and *P. syringae*, *P. nicotianae* was also detected in roots from *E. citridora*; in addition, this species was exclusively detected in roots from *Zelkowa sicula* (Table 1).

#### 3.1.3. Citrus Orchard

**Figure 5:**
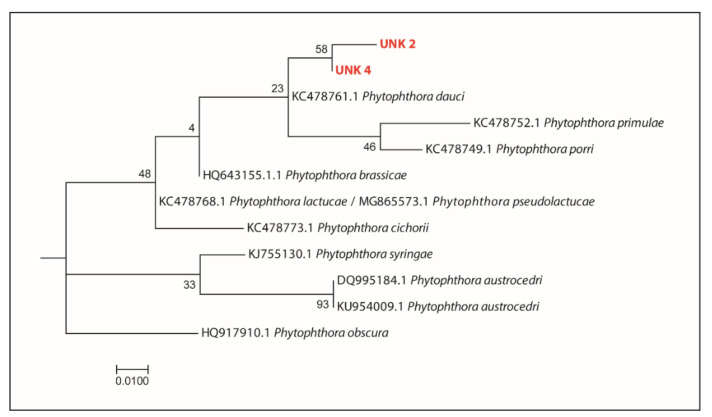
Sections from the phylogenetic tree of the *Phytophthora* ITS1 locus generated by the Maximum Likelihood method, based on the Tamura–Nei model. Position of *Phytophthora unknown* sp.2 and sp.4 within *Phytophthora* clade 8b. The bootstrap consensus tree inferred from 1000 replicates is taken to represent the evolutionary history of the taxa analyzed. Branches corresponding to partitions reproduced in less than 50% bootstrap replicates are collapsed. The percentage of replicate trees in which the associated taxa clustered together in the bootstrap test (1000 replicates) is shown next to the branches.

*P. citrophthora*, *P. nicotianae*, *P. occultans* and two unknown taxa were exclusively identified in roots from the orange trees from the citrus orchard (Table 1 and Table S2). All the detected taxa occurred with a similarly low frequency and barcode read number (Figure 1). Interestingly, two additional unknown *Phytophthora* ASVs, here designated as “*Phytophthora* unknown sp.” 2 (UNK 2) and 4 (UNK 4), were reported from this environment. Phylogenetic analyses placed both ASVs into the *Phytophthora* ITS clade 8b (Figure 5).

#### 3.1.4. Analyses of Biodiversity Resulting from DNA Metabarcoding

The analysis of the biodiversity among *Phytophthora* communities from the three analyzed ecosystems globally evidenced a low evenness from all sites (Table 2).

**Figure 6:**
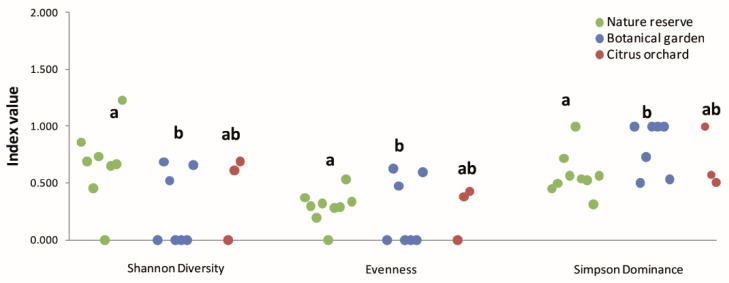
Values of the diversity indices, Shannon diversity, Pielou’s evenness and Simpson dominance, calculated basing on DNA metabarcoding *Phytophthora*-positive rhizosphere soil samples from the three surveyed areas. Data were analyzed with the Kruskal–Wallis test. Different letters indicate significant differences according to Dunn’s multiple comparison tests (*p* ≤ 0.01).

Significant differences in all the values of the indices were reported from the community of the botanical garden, which appears also to be characterized by a very low diversity determined by unbalanced *Phytophthora* populations (Figure 6).

### 3.2. Phytophthora Species Isolated by Baiting

Nineteen out of thirty-nine rhizosphere soil samples (six from the natural reserve, seven from the botanical garden and six from the citrus orchard) processed by baiting revealed the occurrence of *Phytophthora* species from all the surveyed areas. Overall, 122 *Phytophthora* isolates (30 from the nature reserve, 52 from the botanical garden, 40 from the citrus orchard) were obtained. Morphological and ITS sequence analyses made it possible to identify nine *Phytophthora* species (Table 1).

#### 3.2.1. Nature Reserve

Baiting from rhizosphere soil samples from the nature reserve resulted in five *Phytophthora* species from six mature trees belonging to four plant species (Table 1). No more than one *Phytophthora* sp. per sample was obtained. *Phytophthora pseudocryptogea* and *P. cryptogea* were the only species isolated from willow trees (*Salix pedicellata*), *P. bilorbang* was recorded from *Platanus orientalis* and *P. plurivora* from a mature specimen of evergreen oak (*Q. ilex*). Finally, *P. gonapodyides* was isolated from the rhizosphere soil from both *Q. ilex* and *Q. pubescens s. l.*

#### 3.2.2. Botanical Garden

Three *Phytophthora* species were isolated from rhizosphere soil beneath seven out of twelve tree species in the botanical garden (Table 1). *Phytophthora nicotianae* and *P. multivora* occurred together from trees of *Araucaria cookii*, *Phytolacca dioica*, *Q. suber* and *O. europaea*. In addition, *P. multivora* was exclusively isolated from *Sterculia diversifolia* and *Zelkowa sicula*. Finally, *P. parvispora* was isolated from *Coffea arabica*.

#### 3.2.3. Citrus Orchard

*Phytophthora* species recovered by baiting from the citrus orchard comprised *P. nicotianae* and *P. citrophthora*. *nicotianae* was the most prevalent species, occurring from six out of ten sampled trees, while isolates of *P. citrophthora* were obtained from only two trees (Table 1).

### 3.3. Phytophthora-Positive Rhizosphere Soil Samples—Comparison of Outcomes from Baiting and DNA Metabarcoding

The combined application of baiting and DNA metabarcoding made it possible to classify as *Phytophthora*-positive a total of 28 out of the 39 rhizosphere soil samples collected from the three sampling areas; in detail, 10 out of 17 from the nature reserve, 11 out of 12 from the botanical garden and 7 out of 10 from the citrus orchard (Table 1).

**Figure 7:**
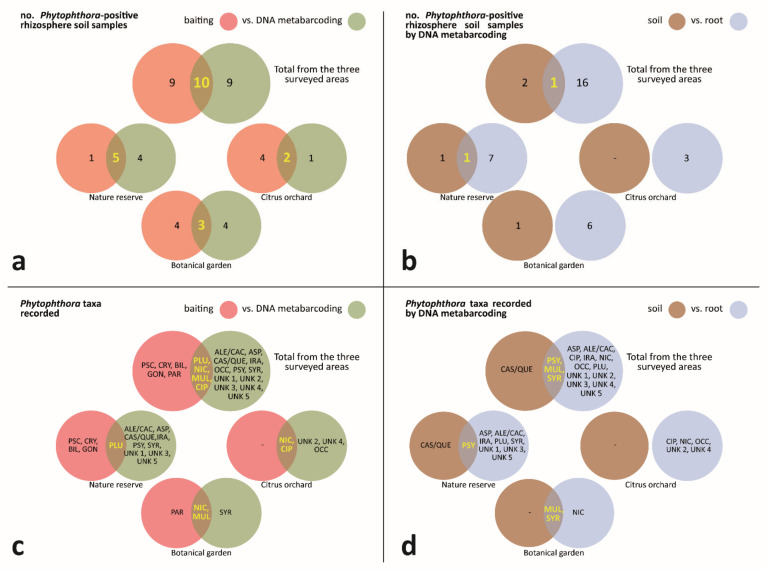
Comparison between: (i) number of *Phytophthora*-positive rhizosphere soil samples by (**a**) baiting and DNA metabarcoding; (**b**) DNA metabarcoding-processing of roots and soil; (ii) *Phytophthora* taxa recorded by (**c**) baiting and DNA metabarcoding; (**d**) DNA metabarcoding-processing of roots and soil. ALE/CAC = *P. aleatoria*/*P. cactorum*; ASP = *P. asparagi*; BIL = *P. bilorbang*; CAS/QUE = *P. castanetorum*/*P. quercina*; CIP = *P. citrophthora*; CRY = *P. cryptogea*; GON = *P. gonapodyides*; IRA-like = *P. iranica*-like; MUL = *P. multivora*; NIC = *P. nicotianae*; OCC = *P. occultans*; PAR = *P. parvispora*; PLU = *P. plurivora*; PSC = *P. pseudocryptogea*; PSY = *P. psychrophila*; SYR = *P. syringae*; UNK 1 = *Phytophthora* unknown sp. 1; UNK 2 = *Phytophthora* unknown sp. 2; UNK 3 = *Phytophthora* unknown sp. 3; UNK 4 = *Phytophthora* unknown sp. 4; UNK 5 = *Phytophthora* unknown sp. 5.

Individually, both techniques showed a similar potential, producing the same number of *Phytophthora*-positive rhizosphere soil samples, namely 19 out of 39, distributed as 9 exclusively by metabarcoding, 9 exclusively by baiting and 10 in common between both techniques (Figure 7a).

With reference to the results from each sampling area, both techniques yielded similar species’ numbers in the botanical garden; in the nature reserve, DNA metabarcoding produced more positives than the baiting; finally, in the citrus orchard, the baiting proved more effective than the DNA metabarcoding (Figure 7a).

#### 3.3.1. Phytophthora-Positive Rhizosphere Soil Samples—Comparison of DNA Metabarcoding Outcomes from Processed Samples of Soil and Root

Among the 19 *Phytophthora*-positive rhizosphere soil samples obtained only by DNA metabarcoding, 2 came exclusively from soil, 16 exclusively from roots, and only 1 in common between roots and soil (Figure 7b). With reference to the results from each sampling area, in the nature reserve the processing of roots generated most positives with only one positive in common with soils; similarly, in the botanical garden and the citrus orchard, there were more positives from root than from soil samples (Figure 7b).

#### 3.3.2. Phytophthora Taxa Recorded—Comparison of Outcomes from Baiting and DNA Metabarcoding

The combined application of baiting and DNA metabarcoding made it possible to record a total of 21 *Phytophthora* taxa, distributed as 5 exclusively by baiting, 12 exclusively by DNA metabarcoding and 4in common between both techniques (Figure 7c). With reference to the results from each sampling area, the techniques were most closely matched in the botanical garden whereas in the nature reserve and the citrus orchard DNA metabarcoding revealed a higher number of species (Figure 7c).

#### 3.3.3. Phytophthora Taxa Recorded—Comparison of DNA Metabarcoding Outcomes from Processed Samples of Soil and Root

Among the 16 *Phytophthora* taxa identified by DNA metabarcoding, only 1 came exclusively from soil, 12 exclusively from roots, and 3 were common to root and soil samples (Figure 7d). With reference to the results from each sampling area, in the nature reserve the processing of roots revealed more taxa, with only *P. psycrophila* in common with soil; in the botanical garden the processing of roots revealed three species with only *P. nicotianae* exclusive to soil; finally, in the citrus orchard, *Phytophthora* taxa were only recorded from processed root samples (Figure 7d).

## 4. Discussion

In this study, 39 composite rhizosphere soil samples collected from three different small-scale ecosystems (17 from a nature reserve, 12 from a botanical garden and 10 from a commercial citrus orchard) in a restricted geographic area of eastern Sicily were processed by both leaf baiting and DNA metabarcoding to investigate the diversity of *Phytophthora* communities. Overall, 72% of the samples tested positive for *Phytophthora* (10 from the nature reserve, 11 from the botanical garden and 7 from the citrus orchard) using both techniques. In total, 1,402,613 *Phytophthora* ITS1 barcode sequence reads (Table S2), together with the 155 *Phytophthora* isolates, made it possible to clearly distinguish 13 known species, 5 unknown *Phytophthora* taxa and 5 that could not be discriminated to a single species (Table 1). Several host plant/*Phytophthora* species binomials identified in this survey are first reports worldwide, including *Phytophthora bilorbang* from *Platanus orientalis* in the nature reserve; *Phytophthora parvispora* from *Coffea arabica; Phytophthora nicotianae* and *P. multivora* from *Araucaria cookie; Phytophthora multivora* from *Phytolacca dioica;* and *Quercus suber*, *Olea europaea* and the IUCN (International Union for Conservation of Nature) protected species *Zelkowa sicula* in the botanical garden. Similarly, several clearly identified *Phytophthora* taxa detected by metabarcoding in this study represent first reports worldwide, including *Phytophthora psychrophila* from *Salix pedicellata* and *Platanus orientalis* in the nature reserve; *Phytophthora syringae* from *Pistacia lentiscus*; *P. syringae* from *Pistacia atlantica* and *Quercus suber* in the botanical garden; as well as *P. occultans* from *Citrus* × *aurantium* in the citrus orchard. *P. occultans* is a homothallic species in ITS clade 2a [84], the same clade encompassing *P. citrophthora*, a pathogen known to be aggressive against citrus [31]. The host range of the latter species includes plant species in multiple families. The relatively high number of new records from the botanical garden confirms that plant diversity conservation sites, such as botanical gardens, arboretums and nature reserves hosting so called ‘sentinel trees’, may have a crucial role in surveillance programs aimed at preventing the introduction of exotic plant pathogens [85,86,87]. Eleven of the 21 recorded *Phytophthora* taxa, including *P. asparagi; P. bilorbang; P. cactorum; P.citrophthora; P. cryptogea; P. multivora; P. nicotianae; P. plurivora; P. pseudocryptogea; P. parvispora* and *P. syringae*, are considered exotic pathogens for European countries [6,37,40,72,78,88,89]. In contrast, *Phytophthora psychrophila* and *P. quercina* are considered endemic to Europe and proposed to originate from species radiation following adaptation to different *Fagaceae* hosts [7,90]. Numerous studies have demonstrated that several horticulturally important *Phytophthora* species are dispersed principally by human-mediated transport of nursery plants [41,88,91,92,93]. The presence and prevalence of presumably exotic species in all three investigated ecosystems in Sicily confirms this assumption. However, it was also shown that the highest values of the diversity indices, Simpson dominance, Shannon diversity and Pielou’s evenness, measuring the diversity of *Phytophthora* species in the targeted ecosystems, were found in the nature reserve. Among the three ecosystems investigated in this study, the nature reserve was the least anthropized one. However, it did comprise many different types of vegetation, suggesting the ecosystem complexity is also a driving factor in shaping the structure of *Phytophthora* soil communities.

In this study, the Illumina metabarcoding of the eDNA and a well-established leaf-baiting isolation technique were applied in parallel to evaluate their potential as detection methods for the description of the variability of *Phytophthora* populations in three different types of small-scale ecosystems. DNA metabarcoding was confirmed as a valuable method to discover the hidden diversity of *Phytophthora* across a range of ecosystems. The reads from the ASV tentatively designated as *P. iranica*-like, deserve particular attention as they did not perfectly match the ex-type of *P. iranica* (differences in 1 bp out of 181 ITS1 bps) and the matches to other available sequences of *Phytophthora* species in the same clade were even lower. Consequently, it is possible that they correspond to *Phytophthora italica*, a validly described species closely related to *P. iranica* with peculiar morphological characteristics, which has not been characterized by DNA sequencing and whose ex-type is no longer available in any culture collection [5,94,95,96]. Further investigation and isolation of living cultures from the same site is required to confirm this hypothesis and fully describe *P. italica*. Among the five unknown *Phytophthora* ASVs, two (*P.* unknown sp. 2 and *P.* unknown sp. 4) were detected in the citrus orchard and both grouped in the 8b clade encompassing host-specific, slow-growing, psychrophilic, homothallic species, infecting herbaceous crops [97,98], and three (*P.* unknown sp. 1, *P.* unknown sp. 3 and *P.* unknown sp. 5), were detected in the nature reserve. Both *P.* unknown sp. 3 and *P.* unknown sp. 5 clustered in the ITS clade 1b, encompassing exclusively homothallic species [96,98,99], while *P.* unknown sp. 1 clustered in the ITS clade 2, a large and rapidly expanding clade in the genus *Phytophthora* [96,98,99], but was not clearly assigned to any of the known subclades of this group. Species in clade 2 are common in both natural habitats and managed ecosystems and many of them are homothallic [28,38,99,100,101]. The submission of these ITS1 sequences and their associated metadata to international DNA databases (Table S1) such as GenBank will contribute to any future field study that is successful in isolating a culture with a corresponding metabarcode. Lastly, in addition to *Phytophthora*, the barcoding primers are known to amplify species of related downy mildew genera [36] and seven such ASVs were detected in samples from the nature reserve and the botanic garden. These ASVs, mostly of unknown species from three different genera, are likely to originate from DNA of airborne downy mildew propagules that have been washed into the soil profile. We acknowledge these barcodes and include recorded reads in Table S3, but they are not discussed further.

In previous studies, high-throughput sequencing methodologies together with baiting techniques were applied in a range of experimental and environmental conditions and in studies with different aims [45,46,64]. In South Africa, the processing by both barcode pyrosequencing and leaf baiting of 120 soil samples, collected from 16 mono-specific plantations of non-native plants and four neighboring natural forests, identified 32 *Phytophthora* phylotypes by metabarcoding, but only five species were identified amongst the 85 isolates recovered and none of the identified species was exclusively detected by baiting [46]. In Italy, as part of a wide sampling campaign carried out in a chestnut forest of the Latium region [45], the leaf baiting of 474 soil samples and the metabarcoding processing of ten of these samples identified 15 *Phytophthora* taxa, which included the nine species also recorded by baiting. In Australia, as part of a monitoring activity of *Phytophthora* populations from five urban parks, the baiting of five rhizosphere soil samples by three different techniques and the metabarcoding analysis was conducted; overall, 30 *Phytophthora* taxa were identified which included only seven species recovered by traditional isolation methods [64].

Consistent with results from South Africa [46], central Italy [45] and Australia [64], in the present study DNA metabarcoding revealed a higher number of *Phytophthora* taxa than baiting. Moreover, only in one sample, BG_1903_S9 from the botanical garden, the same species (*P. multivora*) was simultaneously recorded by both techniques, confirming previous literature [45,46,64]. However, unlike the aforementioned studies, the results from Sicily indicated a relatively high proportion of species detected by baiting that were not detected by DNA metabarcoding, as well as a significantly higher number of metabarcoding-positives from roots over composite soil samples. To explain this latter result, it can be hypothesized that the soil contains substances that interfere with and partly inhibit the DNA amplification process or that the selection of fine roots may be regarded as a form of sample enrichment since mycelium and resting structures (chlamydospores and oospores) of most *Phytophthora* species are strictly associated with living host plant tissues and are not competitive as saprophytes on dead plant material. The latter hypothesis is consistent with the results of Khaliq et al. [64] and Sarker et al. [44] who used enrichment techniques to improve the effectiveness of metabarcoding and baiting in processing composite soil samples. Another possible factor is that the amount of soil/root sample analyzed by metabarcoding is very small compared to that tested by baiting. As a consequence, the probability that rare *Phytophthora* species or species with an uneven distribution in the soil go undetected is high, despite the sensitivity of this diagnostic technique. Enriching the soil sample by selecting the fine roots might be useful to overcome this limitation and increase the accuracy of detection by metabarcoding.

The results of the present study also suggest that the discrepancies between metabarcoding and baiting records could depend, at least in part, on the biology and ecology of the *Phytophthora* species inhabiting the three different targeted ecosystems. It is noticeable that all four identified *Phytophthora* species detected exclusively with DNA metabarcoding, including *P. asparagi*, *P. occultans*, *P. psycrophila* and *P. syringae*, produce thick-walled oospores and are primarily psychrophilic. These features probably allow them to survive and thrive in the Mediterranean climate, characterized by a wide temperature range and long periods of drought [8,35,38,40]. In addition, the five unidentified ASVs detected by DNA metabarcoding clustered in phylogenetic clades encompassing only homothallic species. Quite clearly, baiting provided inconsistent results in recovering *Phytophthora* species producing predominantly this type of resting structures and this could explain why this method failed to detect *P. psycrophila* and *P. syringae,* despite the fact that both species were shown by DNA metabarcoding to be widespread in the nature reserve ecosystem. Conversely, the species recovered by baiting, exclusively or in common with DNA metabarcoding, encompassed both invasive and polyphagous plant pathogens, such as *P. citrophthora; P. cryptogea*; *P. multivora; P. nicotianae; P. parvispora; P. pseudocryptogea* and *P. plurivora*, regardless of their phylogenetic taxonomic position, and species in clade 6, such as *P. bilorbang* and *P. gonapodyides*, which are particularly adapted to an aquatic and saprophytic lifestyle. Both groups share the characteristic of producing a large number of asexual infective propagules (zoospores) [5,15,72,73,78,79] under conducive environmental conditions. This confers upon them the ability to rapidly invade and colonize the ecosystem and, in particular, to be widespread in both natural and anthropized environments and to prevail in managed or disturbed ecosystems. It can be speculated that the experimental conditions during processing of soil samples by baiting favors the recovery of these species. Indeed, during the last few years, studies which tested laboratory protocols for the isolation of *Phytophthora* spp. from soil samples, increasingly highlighted marked differences in achievable outcomes depending on the methodological approach to the baiting [44,102]. It has been observed that both the sensitivity and the variability in *Phytophthora* isolates obtainable by baiting do not merely depend on the quantity and quality of propagules present in the soil, but are affected by technical aspects, such as the size of the baited soil sample, and the baiting container, the use of different species as bait leaves, good practices in limiting the presence of floating organic matter which favors saprotrophic fungal competitors, competition between the *Phytophthora* species present and also the timing of the recovery of infected baits, which leads to the isolation of different species depending on the timing of their zoospore release [44,102].

While metabarcoding is a powerful tool to reveal the diversity of *Phytophthora* populations in environmental samples, other molecular methods, such as microsatellite markers or amplified fragment length polymorphism (AFLP) analysis, must be used to investigate the intraspecific variability of *Phytophthora* [17,18,19,103,104]. An additional limit of metabarcoding is that it provides information on the relative abundance of one taxon compared to others. As a consequence, it is not suitable for quantifying a specific target species and may not be sensitive enough to detect poorly represented taxa, especially when it is based on universal primers. This limit can be overcome by complementing metagenomic with other quantitative detection methods such as multiplex qPCR [105,106].

## 5. Conclusions

In conclusion, while in recent years numerous authors emphasized the high potential of metabarcoding compared to traditional baiting, the present study indicated the complementarity of these two methods in examining the diversity of *Phytophthora* communities in three ecosystems characterized by various levels of anthropization. Moreover, it demonstrated some limitations of each detection method and provided indications of how their combined application will benefit the efficiency and accuracy of surveillance programs in preventing the introduction of exotic invasive species of *Phytophthora* or other pathogens.

## Figures and Tables

**Table 1:** Geographic location of the 39 rhizosphere soil sampling sites from the surveyed natural reserve, botanical garden and citrus orchard, tree species sampled, *Phytophthora* taxa isolated by baiting and identified by DNA metabarcoding.

Sampling Area	Rhizosphere Soil Sample ID.	Location-Country and Geographic Coordinates (DATUM WGS84)	Sampled Tree Species (Baiting/DNA Metabarcoding *Phytophthora*-Positive (+) or Negative (−))	Baited *Phytophthora* Taxa ^1^	DNA Metabarcoding Detected *Phytophthora* Taxa ^1^	*Phytophthora* Spp. (Baiting + DNA Metabarcoding) ^1^
Complesso Speleologico Villasmundo S. Alfio Regional Nature Reserve	NR_1903_S1	Melilli-37°13′17.54″ N; 15°6′19.52″ E	*Salix pedicellata* (+/−)	PSC	-	PSC
	NR_1903_S2	Melilli-37°13′17.66″ N; 15°6′19.28″ E	*S. pedicellata* (+/+)	CRY	PSY (r) ^2^, UNK 3 (r), UNK 5 (r)	CRY, PSY (r), UNK 3 (r), UNK 5 (r)
	NR_1903_S3	Melilli-37°13′17.753″ N; 15°6′18.93″ E	*Platanus orientalis* (−/−)	-	-	-
	NR_1903_S4	Melilli-37°13′17.86″ N; 15°6′18.81″ E	*P. orientalis* (+/+)	BIL	PSY (r), UNK 1 (r)	BIL, PSY (r), UNK 1 (r)
	NR_1903_S5	Melilli-37°13′17.25″ N; 15°6′15.30″ E	*Euphorbia dendroides* (−/−)	-	-	-
	NR_1903_S6	Melilli-37°13′17.48″ N; 15°6′15.31″ E	*Cynara cardunculus* (−/+)	-	IRA-like (r), PSY (r)	IRA-like (r), PSY (r)
	NR_1903_S7	Melilli-37°13′17.60″ N; 15°6′15.30″ E	*Asphodelus* sp. (−/−)	-	-	-
	NR_1903_S8	Melilli-37°13′11.75″ N; 15°6′1.20″ E	*Quercus ilex* (+/+)	GON	CAS/QUE (s) ^2^, PSY (s), SYR (r)	GON, CAS/QUE (s), PSY (s), SYR (r)
	NR_1903_S9	Melilli-37°13′11.00″ N; 15°5′59.69″ E	*Q. ilex* (+/+)	PLU	PSY (s)	PLU, PSY (s)
	NR_1903_S10	Melilli-37°13′10.93″ N; 15°5′59.95″ E	*Q. ilex* (−/−)	-	-	-
	NR_1903_S11	Melilli-37°13′11.788″ N; 15°6′0.547″ E	*Q. pubescens* sensu latu (+/)	GON	PLU (r), PSY (r)	GON, PLU (r), PSY (r)
	NR_1903_S12	Melilli-37°13′17.52″ N; 15°6′7.94″ E	*Sarcopoterium spinosum* (−/−)	-	-	-
	NR_1903_S13	Melilli-37°13′17.50″ N; 15°6′8.57″ E	*S. spinosum* (−/−)	*-*	-	-
	NR_1903_S14	Melilli-37°13′17.28″ N; 15°6′4.77″ E	*Pistacia lentiscus* (−/+)	-	SYR (r), UNK 1 (r)	SYR (r), UNK 1 (r)
	NR_1903_S15	Melilli-37°13′17.50″ N; 15°6′5.13″ E	*P. lentiscus* and *Pyrus* sp., mixed sample (−/−)	-	-	-
	NR_1903_S16	Melilli-37°13′16.94″ N; 15°6′7.66″ E	*P. lentiscus* (−/+)	-	ALE/CAC (r), ASP (r), SYR (r), UNK 1 (r)	ALE/CAC (r), ASP (r), SYR (r), UNK 1 (r)
	NR_1903_S17	Melilli-37°13′16.93″ N; 15°6′6.24″ E	*P. lentiscus* (−/+)	-	ALE/CAC (r), SYR (r), UNK 1 (r)	ALE/CAC (r), SYR (r), UNK 1 (r)
Botanical garden of Catania	BG_1903_S1	Catania-37°30′57.29″ N; 15°5′2.27″ E	*Araucaria cookii* (+/−)	NIC, MUL	-	NIC, MUL
	BG_1903_S2	Catania-37°30′55.92″ N; 15°5′1.95″ E	*Phytolacca dioica* (+/−)	NIC, MUL	-	NIC, MUL
	BG_1903_S3	Catania-37°30′55.08″ N; 15°4′59.75″ E	*Grevillea robusta* (−/+)	-	MUL (r)	MUL (r)
	BG_1903_S4	Catania-37°30′57.56″ N; 15°5′1.47″ E	*Pistacia atlantica* (−/+)	-	MUL (s), SYR (s)	MUL (s), SYR (s)
	BG_1903_S5	Catania-37°30′57.47″ N; 15°5′0.81″ E	*Sterculia diversifolia* (+/−)	MUL	-	MUL
	BG_1903_S6	Catania-37°30′57.69″ N; 15°5′1.80″ E	*Eucalyptus citridora* (−/+)	-	NIC (r), MUL (r), SYR (r)	NIC (r), MUL (r), SYR (r)
	BG_1903_S7	Catania-37°30′53.46″ N; 15°5′2.38″ E	*Zelkowa sicula* (+/+)	MUL	NIC (r)	MUL, NIC (r)
	BG_1903_S8	Catania-37°30′53.35″ N; 15°5′1.89″ E	*Q. suber* (+/+)	NIC, MUL	SYR (r)	NIC, MUL, SYR (r)
	BG_1903_S9	Catania-37°30′53.19″ N; 15°5′2.42″ E	*Olea europea* (+/+)	MUL, NIC	MUL (r)	NIC, MUL, MUL (r)
	BG_1903_S10	Catania-37°30′53.34″ N; 15°5′2.40″ E	*Pistacia lentiscus* (+/)	-	MUL (r), SYR (r)	MUL (r), SYR (r)
	BG_1903_S11	Catania-37°30′57.92″ N; 15°5′0.74″ E	*Coffea arabica* (+/−)	PAR	-	PAR
	BG_1903_S12	Catania-37°30′57.95″ N; 15°5′0.86″ E	*Mangifera indica* (−/−)	-	-	-
Citrus orchard-Tenuta Serravalle	CO_1905_S1	Mineo-37°19′39.38″ N; 14°41′10.65″ E	*Citrus* × *sinensis* ′Tarocco′ nested on *C.* × *aurantium* (−/+)	-	-	-
	CO_1905_S2	Mineo-37°19′39.38″ N; 14°41′10.65″ E	*//*(−/+)	-	NIC (r)	NIC (r)
	CO_1905_S3	Mineo-37°19′40.29″ N; 14°41′11.05″ E	//(+/−)	NIC, CIP	-	NIC, CIP
	CO_1905_S4	Mineo-37°19′40.54″ N; 14°41′12.78″ E	//(+/+)	NIC	UNK 2 (r), UNK 4 (r)	NIC, UNK 2 (r), UNK 4 (r)
	CO_1905_S5	Mineo-37°19′41.35″ N; 14°41′12.90″ E	//(+/+)	NIC	CIP (r), OCC (r)	NIC, CIP (r), OCC (r)
Citrus orchard-Tenuta Serravalle	CO_1905_S6	Mineo-37°19′39.36″ N; 14°41′7.75″ E	//(+/−)	NIC, CIP	-	NIC, CIP
	CO_1905_S7	Mineo-37°19′41.05″ N; 14°41′6.45″ E	//(+/−)	NIC	-	NIC
	CO_1905_S8	Mineo-37°19′41.69″ N; 14°41′7.73″ E	//(+/−)	NIC	-	NIC
	CO_1905_S9	Mineo-37°19′41.97″ N; 14°41′9.30″ E	//(−/−)	-	-	-
	CO_1905_S10	Mineo-7°19′43.16″ N; 14°41′7.12″ E	//(−/−)	-	-	-

^1^ ALE/CAC = *P. aleatoria*/*P. cactorum*; ASP = *P. asparagi*; BIL = *P. bilorbang*; CAS/QUE = *P. castanetorum*/*P. quercina*; CIP = *P. citrophthora*; CRY = *P. cryptogea*; GON = *P. gonapodyides*; IRA-like = *P. iranica-like*; MUL = *P. multivora*; NIC = *P. nicotianae*; OCC = *P. occultans*; PAR = *P. parvispora*; PLU = *P. plurivora*; PSC = *P. pseudocryptogea*; PSY = *P. psychrophila*; SYR = *P. syringae*; UNK 1 = *Phytophthora* unknown sp. 1; UNK 2 = *Phytophthora* unknown sp. 2; UNK 3 = *Phytophthora* unknown sp. 3; UNK 4 = *Phytophthora* unknown sp. 4; UNK 5 = *Phytophthora* unknown sp. 5; ^2^ matrix of detection: r = roots; s = soil.

**Table 2:** Comparison of diversity indices of the *Phytophthora* community in DNA metabarcoding rhizosphere soil-positive samples from the three surveyed areas. Data were analyzed with the Kruskal–Wallis test.

Sampling Area			
Diversity Index	Nature Reserve	Botanical Garden	Citrus Orchard
Shannon diversity	0.674 a	0.267 b	0.436 ab
Pielou’s evenness	0.29 a	0.24 b	0.27 ab
Simpson dominance	0.57 a	0.82 b	0.69 ab

Different letters indicate significant differences according to Dunn’s multiple comparison tests (*p* ≤ 0.01).

## Data Availability

Data are contained within Supplementary Material GenBank accession numbers of ITS sequences of representative isolates obtained in this study are available in Table S1. Data are available in a publicly accessible repository. The raw Illumina data presented in this study are openly available in Zenodo at [107] as FASTQ files.

## Supplementary Materials

The following are available online at https://www.mdpi.com/article/10.3390/jof8040330/s1, Table S1: Isolation details and GenBank accession numbers of representative *Phytophthora* isolates obtained by baiting of rhizosphere soil samples from the three sampling areas; Table S2: *Phytophthora* species identified by the Amplicon Sequence Variants (ASVs) recorded in this study, and match ranked by number of reads across the *Phytophthora*-positive samples from three surveyed areas in Sicily; Table S3. Additional oomycete taxa recognized by Amplicon Sequence Variants (AVSs) recorded in this study, and match ranked by number of reads across the Illumina-positive samples from three surveyed areas in Sicily.
