# Supplementary material for: DNA Metabarcoding and Isolation by Baiting Complement Each Other in Revealing Phytophthora Diversity in Anthropized and Natural Ecosystems
Source: J Fungi (Basel). 2022 Mar 22;8(4):330. doi: 10.3390/jof8040330 (PMC9028584; doi:10.3390/jof8040330)
Supplement: Supplementary file 1 [file jof-08-00330-s001.zip › jof-1647935-supplementary.pdf]

Table S1. Isolation details and GenBank accession numbers of representative *Phytophthora* isolates obtained by baiting of rhizosphere soil samples from the three sampling areas.

***Phytophthora* species**	**Isolate code**	**Host**	**Sampling area and Municipality (City, Province, State)**	**Kind of ecosystem**	**Isolation date**	**GenBank accession no. of ITS sequences**
P. bilorbang	I-3K2A	*Platanus orientalis*	Complesso Speleologico Villasmundo - S. Alfio RNR (Melilli, Siracusa, Italy)	Natural	March-2019	OL679976
P. citrophthora	SER_3_2K1a	*Citrus × sinensis* var. navelina	Citrus Orchard - Tenuta Serravalle (Mineo, Catania, Italy)	Agricultural	May-2019	OL679977
P. cryptogea	I-2K19C	*Salix pedicellata*	Complesso Speleologico Villasmundo - S. Alfio RNR (Melilli, Siracusa, Italy)	Natural	March-2019	OL679978
	I-2K13A	*S. pedicellata*	Complesso Speleologico Villasmundo - S. Alfio RNR (Melilli, Siracusa, Italy)	Natural	March-2019	OL679979
P. gonapodyides	IV-26A	*Quercus ilex*	Complesso Speleologico Villasmundo - S. Alfio RNR (Melilli, Siracusa, Italy)	Natural	March-2019	OL679980
P. multivora	VII-1K1B	*Olea europea*	Botanical Garden of Catania (Catania, Italy)	Semi-natural	April-2019	OL679981
	IX-1K5A	*Sterculia diversifolia*	Botanical Garden of Catania (Catania, Italy)	Semi-natural	April-2019	OL679982
	X-1K4A	*Phytolacca dioica*	Botanical Garden of Catania (Catania, Italy)	Semi-natural	April-2019	OL679983
	V-1K1A	*Araucaria cookii*	Botanical Garden of Catania (Catania, Italy)	Semi-natural	April-2019	OL679984
	II-1K8A	*Zelkova sicula*	Botanical Garden of Catania (Catania, Italy)	Semi-natural	April-2019	OL679985
	XII-1K1A	*Q. suber*	Botanical Garden of Catania (Catania, Italy)	Semi-natural	April-2019	OL679986
P. nicotianae	VII-1K3D	*O. europea*	Botanical Garden of Catania (Catania, Italy)	Semi-natural	April-2019	OL679987
	V-1K6A	*A. cookii*	Botanical Garden of Catania (Catania, Italy)	Semi-natural	April-2019	OL679988
	XII-1K2A	*Q. suber*	Botanical Garden of Catania (Catania, Italy)	Semi-natural	April-2019	OL679989
	X-1K8A	*P. dioica*	Botanical Garden of Catania (Catania, Italy)	Semi-natural	April-2019	OL679990
	SER_3_1K4b	*C. × sinensis* Tarocco /*C. × aurantium* rootstock	Citrus Orchard - Tenuta Serravalle (Mineo, Catania, Italy)	Agricultural	May-2019	OL679991
P. parvispora	IV-1K3A	*Coffea arabica*	Botanical Garden of Catania (Catania, Italy)	Semi-natural	April-2019	OL679992
P. plurivora	IV-4K9D	*Q. ilex*	Complesso Speleologico Villasmundo - S. Alfio RNR (Melilli, Siracusa, Italy)	Natural	March-2019	OL679993

Cont. Table S1

***Phytophthora* species**	**Isolate code**	**Host**	**Sampling area and Municipality (City, Province, State)**	**Kind of ecosystem**	**Isolation date**	**GenBank accession no. of ITS sequences**
P. plurivora	IV-4K14B	*Q. ilex*	Complesso Speleologico Villasmundo - S. Alfio RNR (Melilli, Siracusa, Italy)	Natural	March-2019	OL679994
P. pseudocryptogea	I-1K10A	*S. pedicellata*	Complesso Speleologico Villasmundo - S. Alfio RNR (Melilli, Siracusa, Italy)	Natural	March-2019	OL679995
	I-1K11A	*S. pedicellata*	Complesso Speleologico Villasmundo - S. Alfio RNR (Melilli, Siracusa, Italy)	Natural	March-2019	OL679996
	I-1K5A	*S. pedicellata*	Complesso Speleologico Villasmundo - S. Alfio RNR (Melilli, Siracusa, Italy)	Natural	March-2019	OL679997

Table S2. *Phytophthora* species identified by the Amplicon Sequence Variants (AVSs) recorded in this study, and match ranked by number of reads across the *Phytophthora*-positive samples from three surveyed areas in Sicily.

		No. reads per type of *Phytophthora*-positive sample (S = soil / R = roots) from the Nature Reserve (sample code NR_1903_S"")																		No. reads per type of *Phytophthora*-positive sample (S = soil / R = roots) from the Botanical garden (sample code BG_1903_S"")														No. reads per type of *Phytophthora*-positive sample (S = soil / R = roots) from the Citrus orchard (sample code CO_1905_S"")							
		2		4		6		8		9		11		14		16		17		3		4		6		7		8		9		10		2		4		5		Total n. of reads per species	
**Identified species**	**no. different associated OTUs**	S	R	S	R	S	R	S	R	S	R	S	R	S	R	S	R	S	R	S	R	S	R	S	R	S	R	S	R	S	R	S	R	S	R	S	R	S	R	S	R	S	R
P. asparagi	1	-	-	-	-	-	-	-	-	-	-	-	-	-	-	-	9130	-	-	-	-	-	-	-	-	-	-	-	-	-	-	-	-	-	-	-	-	-	-	-	-	-	9130
P. aleatoria / *P. cactorum*	1	-	-	-	-	-	-	-	-	-	-	-	-	-	-	-	7848	-	6272	-	-	-	-	-	-	-	-	-	-	-	-	-	-	-	-	-	-	-	-	-	-	-	14120
P. castanetorum / *P. quercina*	1	-	-	-	-	-	-	46798	-	-	-	-	-	-	-	-	-	-	-	-	-	-	-	-	-	-	-	-	-	-	-	-	-	-	-	-	-	-	-	-	-	46798	-
P. citrophthora	2	-	-	-	-	-	-	-	-	-	-	-	-	-	-	-	-	-	-	-	-	-	-	-	-	-	-	-	-	-	-	-	-	-	-	-	-	-	-	-	54296	-	54296
P. iranica-like	1	-	-	-	-	-	2609	-	-	-	-	-	-	-	-	-	-	-	-	-	-	-	-	-	-	-	-	-	-	-	-	-	-	-	-	-	-	-	-	-	-	-	2609
P. multivora	2	-	-	-	-	-	-	-	-	-	-	-	-	-	-	-	-	-	-	-	72000	20098	-	-	60173	-	-	-	-	-	43584	-	75790	-	-	-	-	-	-	-	-	20098	251547
P. nicotianae	7	-	-	-	-	-	-	-	-	-	-	-	-	-	-	-	-	-	-	-	-	-	-	-	7252	-	78597	-	-	-	-	-	-	-	-	43176	-	-	-	-	-	-	129025
P. occultans	1	-	-	-	-	-	-	-	-	-	-	-	-	-	-	-	-	-	-	-	-	-	-	-	-	-	-	-	-	-	-	-	-	-	-	-	-	-	-	-	48145	-	48145
P. plurivora	1	-	-	-	-	-	-	-	-	-	-	-	21062	-	-	-	-	-	-	-	-	-	-	-	-	-	-	-	-	-	-	-	-	-	-	-	-	-	-	-	-	-	21062
P. psychrophila	1	-	29730	-	22741	-	12799	222235	-	96225	-	-	37623	-	-	-	-	-	-	-	-	-	-	-	-	-	-	-	-	-	-	-	-	-	-	-	-	-	-	-	-	318460	102893
P. syringae	1	-	-	-	-	-	-	-	9898	-	-	-	-	-	29727	-	39695	-	30928	-	-	24122	-	-	3569	-	-	-	26792	-	-	-	44575	-	-	-	-	-	-	-	-	24122	185184
P. unknown sp. 1	1	-	-	-	22469	-	-	-	-	-	-	-	-	-	18547	-	23668	-	5297	-	-	-	-	-	-	-	-	-	-	-	-	-	-	-	-	-	-	-	-	-	-	-	69981
P. unknown sp. 2	1	-	-	-	-	-	-	-	-	-	-	-	-	-	-	-	-	-	-	-	-	-	-	-	-	-	-	-	-	-	-	-	-	-	-	-	51327	-	-	-	-	-	51327
P. unknown sp. 3	1	-	28032	-	-	-	-	-	-	-	-	-	-	-	-	-	-	-	-	-	-	-	-	-	-	-	-	-	-	-	-	-	-	-	-	-	-	-	-	-	-	-	28032
P. unknown sp. 4	1	-	-	-	-	-	-	-	-	-	-	-	-	-	-	-	-	-	-	-	-	-	-	-	-	-	-	-	-	-	-	-	-	-	-	-	22591	-	-	-	-	-	22591
P. unknown sp. 5	1	-	3193	-	-	-	-	-	-	-	-	-	-	-	-	-	-	-	-	-	-	-	-	-	-	-	-	-	-	-	-	-	-	-	-	-	-	-	-	-	-	-	3193

Table S3. Additional oomycete taxa recognized by Amplicon Sequence Variants (AVSs) recorded in this study, and match ranked by number of reads across the Illumina-positive samples from three surveyed areas in Sicily.

	No. reads of per type of Illumina-positive sample (S = soil / R = roots) from the Nature Reserve (sample code NR_1903_S"")								No. reads of per type of Illumina-positive sample (S = soil / R = roots) from the Botanical Garden (sample code BG_1903_S"")		No. reads of per type of Illumina-positive sample (S = soil / R = roots) from the Citrus Orchard						Total n. of reads per species	
	6		7		8		9		6		NO POSITIVE SAMPLES							
Identified taxon (% identity)	S	R	S	R	S	R	S	R	S	R							S	R
Peronospora elucastri (100%)	-	56085	-	-	-	-	-	-	-	-	-	-	-	-	-	-	-	56085
Peronospora viciae	-	-	-	-	-	-	2783	-	-	-	-	-	-	-	-	-	2783	
Plasmopara sp. (98.19%)	-	-	16220	-	-	-	-	-	-	-	-	-	-	-	-	-	16220	-
Plasmopara sp. (98.19%)	-	-	10946	-	-	-	-	-	-	-	-	-	-	-	-	-	10946	-
Hyaloperonospora sp. (99.52)	-	-	-	-	-	-	-	-	-	4668	-	-	-	-	-	-	-	4668
Peronospora sp. (99.46%)	-	4585	-	-	-	-	-	-	-	-	-	-	-	-	-	-	-	4585
Hyaloperonospora lunariae (100%)	-	3458	-	-	-	-	-	-	-	-	-	-	-	-	-	-	-	3458
Phytophthora psycrophila variant (99.43%)	-	-	-	-	2561	-	-	-	-	-	-	-	-	-	-	-	2561	-
